# Supplementary material for: Computation suggests that the cell adhesion sub-proteome is enriched for sites of pH-dependence and charge burial
Source: PLoS One. 2025 Dec 9;20(12):e0338186. doi: 10.1371/journal.pone.0338186 (PMC12688153; doi:10.1371/journal.pone.0338186)
Supplement: S1 Table — (PDF) [file pone.0338186.s001.pdf]

S1 Table. Detailed filter performance.

filter method[a]	*SASA*[a]	*pH*[b] *%*	*bm*[c] */ 23*	*enrich*[b] *ET-NET*	*sens* [d]	*spec* [d]	*bal acc*[d]	*chosen filter name*[a]
pkcalc-int-3	25	81.4	17	2.81	0.74	0.74	0.74	
	20	77.8	16	2.99	0.69	0.77	0.73	
	**15**	**71.6**	**16**	**3.25**	**0.63**	**0.81**	**0.72**	**pkcalc-int-15-3**
	10	62.4	14	3.58	0.53	0.85	0.69	
	5	48.4	9	4.33	0.38	0.91	0.65	
	3	40.5	8	4.99	0.30	0.94	0.62	
pkcalc-int-2	25	94.8	22	1.91	0.91	0.52	0.72	
	20	94.0	21	2.05	0.89	0.57	0.73	
	**15**	**91.2**	**20**	**2.24**	**0.85**	**0.62**	**0.74**	**pkcalc-int-15-2**
	10	85.0	18	2.51	0.79	0.68	0.74	
	**5**	**75.8**	**16**	**2.96**	**0.68**	**0.77**	**0.72**	**pkcalc-int-5-2**
	3	67.0	15	3.32	0.59	0.82	0.71	
propka-range	25	93.5	20	1.79	0.91	0.49	0.70	
	20	93.1	20	1.90	0.89	0.53	0.71	
	**15**	**90.5**	**20**	**2.08**	**0.85**	**0.59**	**0.72**	**propka-range-15**
	10	84.0	18	2.36	0.79	0.66	0.73	
	**5**	**75.2**	**18**	**2.66**	**0.69**	**0.74**	**0.72**	**propka-range-5**
	3	70.0	13	2.84	0.63	0.78	0.70	
pkcalc-range	25	85.6	19	1.71	0.82	0.52	0.67	
	20	82.4	19	1.78	0.78	0.56	0.67	
	15	77.5	18	1.87	0.72	0.61	0.67	
	10	69.0	16	2.01	0.63	0.69	0.66	
	5	58.2	14	2.17	0.50	0.77	0.63	
	3	47.4	10	2.22	0.42	0.81	0.62	
intersection	**15**	**95.8**	**17**	**1.85**	**0.93**	**0.50**	**0.71**	**pkcalc-15-2-propka-15**
intersection	**15**	**93.1**	**15**	**2.03**	**0.89**	**0.56**	**0.72**	**pkcalc-15-3-propka-15**
intersection	**5**	**85.0**	**14**	**2.42**	**0.80**	**0.67**	**0.74**	**pkcalc-5-2-propka-5**
pkcalc-int-nodh-3	**n/a**	**65.0**	**9**	**4.34**	**0.54**	**0.88**	**0.71**	**pkcalc-int-nodh-3**
pkcalc-deltaQ-10	**10**	**63.1**	**14**	**2.49**	**0.50**	**0.80**	**0.65**	**pkcalc-deltaQ-10**

S1 Table Footnotes: [a]SASA thresholds (Å^2) determine extent of burial required, and relate to plots in Fig 1, along with the filter method and filters chosen for further analysis (in bold). [b]pH and enrich ET-NET relate to the ordinates of the plots in Fig 1, where pH % is the % of proteins in the known pH-dependent subset (306 total) that are predicted to be pH-dependent. [c]The number of the benchmark set of proteins (23 total) predicted to be pH-dependent by each filter is given. [d]Whereas the pH-dependent and benchmark subsets cannot be analysed with sensitivity/specificity, since other members of the population are not classified, the ET and NET subsets are largely established, and analysed (sens, spec), along with the balanced accuracy.
