# Supplementary material for: Computation suggests that the cell adhesion sub-proteome is enriched for sites of pH-dependence and charge burial
Source: PLoS One. 2025 Dec 9;20(12):e0338186. doi: 10.1371/journal.pone.0338186 (PMC12688153; doi:10.1371/journal.pone.0338186)
Supplement: S1 Fig — Heat map of filter results for any GO category that has ≥ 3-fold enrichment for any filter calculation. (PDF) [file pone.0338186.s002.pdf]

GO-ID	GO-NAME	pkcalc-int-15-2	pkcalc-int-nodh-3	pkcalc-int-15-3	pkcalc-int-5-2	propka-range-15	propka-range-5	pkcalc-15-3-propka-15	pkcalc-15-2-propka-15	pkcalc-5-2-propka-5	pkcalc-deltaQ-10
GO:0021637	trigeminal nerve structural organization										
GO:0021636	trigeminal nerve morphogenesis										
GO:0007193	adenylate cyclase-inhibiting G protein-coupled r										
GO:0007186	G protein-coupled receptor signaling pathway										
GO:0098664	G protein-coupled serotonin receptor signaling p										
GO:0007196	adenylate cyclase-inhibiting G protein-coupled g										
GO:0007216	G protein-coupled glutamate receptor signaling										
GO:0007215	glutamate receptor signaling pathway										
GO:0007160	cell-matrix adhesion										
GO:0032483	regulation of Rab protein signal transduction										
GO:0051056	regulation of small GTPase mediated signal trans										
GO:0050955	thermoception										
GO:0007600	sensory perception										
GO:0050906	detection of stimulus involved in sensory percep										
GO:0051606	detection of stimulus										
GO:0032253	dense core granule localization										
GO:0032252	secretory granule localization										
GO:0007043	cell-cell junction assembly										
GO:0034329	cell junction assembly										
GO:0015793	glycerol transmembrane transport										
GO:0098609	cell-cell adhesion										
GO:0086001	cardiac muscle cell action potential										
GO:0001508	action potential										
GO:0051899	membrane depolarization										
GO:0009593	detection of chemical stimulus										
GO:0035589	G protein-coupled purinergic nucleotide recepto										
GO:0035590	purinergic nucleotide receptor signaling pathway										
GO:0050911	detection of chemical stimulus involved in senso										
GO:0050907	detection of chemical stimulus involved in senso										
GO:0007606	sensory perception of chemical stimulus										
GO:0007608	sensory perception of smell										
GO:0016339	calcium-dependent cell-cell adhesion via plasma										
GO:0098742	cell-cell adhesion via plasma-membrane adhesic										
GO:0007187	G protein-coupled receptor signaling pathway co										
GO:0099505	regulation of presynaptic membrane potential										
GO:0140627	ubiquitin-dependent protein catabolic process v										
GO:0051016	barbed-end actin filament capping										
GO:1904816	positive regulation of protein localization to chro										
GO:0097104	postsynaptic membrane assembly										
GO:0007416	synapse assembly										
GO:0007200	phospholipase C-activating G protein-coupled re										
GO:0030048	actin filament-based movement										
GO:0044331	cell-cell adhesion mediated by cadherin										
GO:0007214	gamma-aminobutyric acid signaling pathway										
GO:0045761	regulation of adenylate cyclase activity										
GO:0034332	adherens junction organization										
GO:0071880	adenylate cyclase-activating adrenergic receptor										
GO:0071875	adrenergic receptor signaling pathway										
GO:0090630	activation of GTPase activity										
GO:0001755	neural crest cell migration										
GO:0090497	mesenchymal cell migration										
GO:0014032	neural crest cell development										
GO:0050919	negative chemotaxis										
GO:0007156	homophilic cell adhesion via plasma membrane										
GO:1990806	ligand-gated ion channel signaling pathway										
GO:0071526	semaphorin-plexin signaling pathway										
GO:0032482	Rab protein signal transduction										
GO:0007190	activation of adenylate cyclase activity										
GO:0045762	positive regulation of adenylate cyclase activity										
GO:0051932	synaptic transmission GABAergic										
GO:0086002	cardiac muscle cell action potential involved in co										
GO:0086003	cardiac muscle cell contraction										
GO:0070252	actin-mediated cell contraction										
GO:1904862	inhibitory synapse assembly										
GO:0007271	synaptic transmission cholinergic										
GO:0035025	positive regulation of Rho protein signal transdu										
GO:0051057	positive regulation of small GTPase mediated sig										
GO:0035023	regulation of Rho protein signal transduction										
GO:0034472	snRNA 3'-end processing										
GO:0016180	snRNA processing										
GO:0019228	neuronal action potential										
GO:0033627	cell adhesion mediated by integrin										
GO:0098703	calcium ion import across plasma membrane										
GO:0070509	calcium ion import										
GO:1902656	calcium ion import into cytosol										
GO:0006833	water transport										
GO:0007229	integrin-mediated signaling pathway										
