# Supplementary material for: Computation suggests that the cell adhesion sub-proteome is enriched for sites of pH-dependence and charge burial
Source: PLoS One. 2025 Dec 9;20(12):e0338186. doi: 10.1371/journal.pone.0338186 (PMC12688153; doi:10.1371/journal.pone.0338186)
Supplement: S2 Fig — Heat map of filter results for any GO category that has ≥ 3-fold enrichment for any filter calculation. (PDF) [file pone.0338186.s003.pdf]

GO-ID	GO-NAME	pkcalc-int-15-2	pkcalc-int-nadh-3	pkcalc-int-15-3	pkcalc-int-5-2	propka-range-15	propka-range-5	pkcalc-15-3-propka-15	pkcalc-15-2-propka-15	pkcalc-5-2-propka-5	pkcalc-deltaQ-10
GO:0004888	transmembrane signaling receptor activity										
GO:0038023	signaling receptor activity										
GO:0060089	molecular transducer activity										
GO:0016494	C-X-C chemokine receptor activity										
GO:0004950	chemokine receptor activity										
GO:0001637	G protein-coupled chemoattractant receptor act										
GO:0004930	G protein-coupled receptor activity										
GO:0016493	C-C chemokine receptor activity										
GO:0016502	nucleotide receptor activity										
GO:0022829	wide pore channel activity										
GO:0005243	gap junction channel activity										
GO:0001640	adenylate cyclase inhibiting G protein-coupled gl										
GO:0098988	G protein-coupled glutamate receptor activity										
GO:0008066	glutamate receptor activity										
GO:0004983	neuropeptide Y receptor activity										
GO:0008188	neuropeptide receptor activity										
GO:0008528	G protein-coupled peptide receptor activity										
GO:0001653	peptide receptor activity										
GO:0005267	potassium channel activity										
GO:0005227	calcium-activated cation channel activity										
GO:0022839	monoatomic ion-gated channel activity										
GO:0005245	voltage-gated calcium channel activity										
GO:0022843	voltage-gated monoatomic cation channel activi										
GO:0005244	voltage-gated monoatomic ion channel activity										
GO:0022832	voltage-gated channel activity										
GO:0045125	bioactive lipid receptor activity										
GO:0001594	trace-amine receptor activity										
GO:0008227	G protein-coupled amine receptor activity										
GO:0022851	GABA-gated chloride ion channel activity										
GO:0022824	transmitter-gated monoatomic ion channel activ										
GO:0005230	extracellular ligand-gated monoatomic ion chan										
GO:0022835	transmitter-gated channel activity										
GO:0030594	neurotransmitter receptor activity										
GO:0004970	glutamate-gated receptor activity										
GO:0099604	ligand-gated calcium channel activity										
GO:0022848	acetylcholine-gated monoatomic cation-selective										
GO:1904315	transmitter-gated monoatomic ion channel activ										
GO:0099529	neurotransmitter receptor activity involved in re										
GO:0098960	postsynaptic neurotransmitter receptor activity										
GO:0005231	excitatory extracellular ligand-gated monoatomi										
GO:0008569	minus-end-directed microtubule motor activity										
GO:0003777	microtubule motor activity										
GO:0003774	cytoskeletal motor activity										
GO:0033038	bitter taste receptor activity										
GO:0008527	taste receptor activity										
GO:0019956	chemokine binding										
GO:0008331	high voltage-gated calcium channel activity										
GO:0070679	inositol 145 trisphosphate binding										
GO:0005549	odorant binding										
GO:1901338	catecholamine binding										
GO:0005272	sodium channel activity										
GO:0015254	glycerol channel activity										
GO:0015168	glycerol transmembrane transporter activity										
GO:0016500	protein-hormone receptor activity										
GO:0004993	G protein-coupled serotonin receptor activity										
GO:0004953	icosanoid receptor activity										
GO:0016907	G protein-coupled acetylcholine receptor activity										
GO:0099528	G protein-coupled neurotransmitter receptor ac										
GO:0015464	acetylcholine receptor activity										
GO:0004935	adrenergic receptor activity										
GO:0004936	alpha-adrenergic receptor activity										
GO:0098639	collagen binding involved in cell-matrix adhesion										
GO:0005249	voltage-gated potassium channel activity										
GO:0005223	intracellularly cGMP-activated cation channel ac										
GO:0005221	intracellularly cyclic nucleotide-activated monoa										
GO:0043855	cyclic nucleotide-activated monoatomic ion chan										
GO:0005217	intracellularly ligand-gated monoatomic ion chan										
GO:0005222	intracellularly cAMP-activated cation channel act										
GO:0045028	G protein-coupled purinergic nucleotide recepto										
GO:0001614	purinergic nucleotide receptor activity										
GO:0004890	GABA-A receptor activity										
GO:0016917	GABA receptor activity										
GO:0004984	olfactory receptor activity										
GO:0019957	C-C chemokine binding										
GO:0030215	semaphorin receptor binding										
GO:0008574	plus-end-directed microtubule motor activity										
GO:0042923	neuropeptide binding										
GO:0015250	water channel activity										
GO:0005372	water transmembrane transporter activity										
GO:0008020	G protein-coupled photoreceptor activity										
GO:0009881	photoreceptor activity										
GO:0017154	semaphorin receptor activity										
GO:0008503	benzodiazepine receptor activity										
GO:0051378	serotonin binding										
GO:0061608	nuclear import signal receptor activity										
GO:0140142	nucleocytoplasmic carrier activity										
GO:0004955	prostaglandin receptor activity										
GO:0004954	prostanoid receptor activity										
GO:0000146	microfilament motor activity										
GO:0005544	calcium-dependent phospholipid binding										
GO:0005545	1-phosphatidylinositol binding										
GO:0005085	guanyl-nucleotide exchange factor activity										
GO:0030695	GTPase regulator activity										
GO:0060589	nucleoside-triphosphatase regulator activity										
GO:0005096	GTPase activator activity										
GO:0008381	mechanosensitive monoatomic ion channel activ										
GO:0005251	delayed rectifier potassium channel activity										
GO:0005041	low-density lipoprotein particle receptor activity										
GO:0045294	alpha-catenin binding										
GO:0098973	structural constituent of postsynaptic actin cytos										
GO:0003707	nuclear steroid receptor activity										
GO:0005248	voltage-gated sodium channel activity										
GO:1990756	ubiquitin-like ligase-substrate adaptor activity										
GO:0140767	enzyme-substrate adaptor activity										
GO:0140662	ATP-dependent protein folding chaperone										
GO:0051015	actin filament binding										
GO:0045499	chemorepellent activity										
GO:0030020	extracellular matrix structural constituent confer										
GO:0005547	phosphatidylinositol-345-trisphosphate binding										
GO:0008013	beta-catenin binding										
GO:0032934	sterol binding										
