# Supplementary material for: Computation suggests that the cell adhesion sub-proteome is enriched for sites of pH-dependence and charge burial
Source: PLoS One. 2025 Dec 9;20(12):e0338186. doi: 10.1371/journal.pone.0338186 (PMC12688153; doi:10.1371/journal.pone.0338186)
Supplement: Attachment — Submitted filename: revision-notes.docx [file pone.0338186.s006.docx]

PONE-D-25-42837
Computational investigation suggests that the cell adhesion sub-proteome is enriched for sites of pH-dependence and charge burial, whilst some key
intracellular pathways may be shielded from pH fluctuations.
PLOS ONE
Dear Dr. Warwicker,
Thank you for submitting your manuscript to PLOS ONE. After careful consideration, we feel that it has merit but does not fully meet PLOS ONE’s
publication criteria as it currently stands. Therefore, we invite you to submit a revised version of the manuscript that addresses the points raised during the
review process.
==============================
Please carefully address the reviewer's comments. The response will be re-reviewed by the same reviewers. Both reviewers raised concerns on the modeling,
which is one major point that need to be carefully addressed. Additional analysis and/or data may be required to fully address the critic regarding model/data
validity.
==============================
Please submit your revised manuscript by Oct 19 2025 11:59PM. If you will need more time than this to complete your revisions, please reply to this message
or contact the journal office at plosone@plos.org. When you're ready to submit your revision, log on to https://www.editorialmanager.com/pone/
[editorialmanager.com] and select the 'Submissions Needing Revision' folder to locate your manuscript file.
Please include the following items when submitting your revised manuscript:
• A rebuttal letter that responds to each point raised by the academic editor and reviewer(s). You should upload this letter as a separate file labeled
'Response to Reviewers'.
• A marked-up copy of your manuscript that highlights changes made to the original version. You should upload this as a separate file labeled 'Revised
Manuscript with Track Changes'.
• An unmarked version of your revised paper without tracked changes. You should upload this as a separate file labeled 'Manuscript'.
2
If you would like to make changes to your financial disclosure, please include your updated statement in your cover letter. Guidelines for resubmitting your
figure files are available below the reviewer comments at the end of this letter.
If applicable, we recommend that you deposit your laboratory protocols in protocols.io to enhance the reproducibility of your results. Protocols.io assigns your
protocol its own identifier (DOI) so that it can be cited independently in the future. For instructions see: https://journals.plos.org/plosone/s/submissionguidelines#loc-laboratory-protocols [track.editorialmanager.com]. Additionally, PLOS ONE offers an option for publishing peer-reviewed Lab Protocol
articles, which describe protocols hosted on protocols.io. Read more information on sharing protocols at https://plos.org/protocols?utm_medium=editorialemail&utm_source=authorletters&utm_campaign=protocols [track.editorialmanager.com].
We look forward to receiving your revised manuscript.
Kind regards,
Xiangming Zha, Ph.D.
Academic Editor
PLOS ONE
Journal Requirements:

When submitting your revision, we need you to address these additional requirements.
1. Please ensure that your manuscript meets PLOS ONE's style requirements, including those for file naming. The PLOS ONE style templates can be found at
https://journals.plos.org/plosone/s/file?id=wjVg/PLOSOne_formatting_sample_main_body.pdf [journals.plos.org] and
https://journals.plos.org/plosone/s/file?id=ba62/PLOSOne_formatting_sample_title_authors_affiliations.pdf [track.editorialmanager.com]
2. Thank you for stating the following financial disclosure:
“This work was supported by UK Biotechnology and Biological Sciences Research Council grant BB/V0065921/1 to JW”
Please state what role the funders took in the study. If the funders had no role, please state: "The funders had no role in study design, data collection and
analysis, decision to publish, or preparation of the manuscript."
If this statement is not correct you must amend it as needed.
Please include this amended Role of Funder statement in your cover letter; we will change the online submission form on your behalf.
3

3. We note that there is identifying data in the Supporting Information file < S1-File.xlsx>. Due to the inclusion of these potentially identifying data, we have
removed this file from your file inventory. Prior to sharing human research participant data, authors should consult with an ethics committee to ensure data are
shared in accordance with participant consent and all applicable local laws.
Data sharing should never compromise participant privacy. It is therefore not appropriate to publicly share personally identifiable data on human research
participants. The following are examples of data that should not be shared:
-Name, initials, physical address
-Ages more specific than whole numbers
-Internet protocol (IP) address
-Specific dates (birth dates, death dates, examination dates, etc.)
-Contact information such as phone number or email address
-Location data
-ID numbers that seem specific (long numbers, include initials, titled “Hospital ID”) rather than random (small numbers in numerical order)
Data that are not directly identifying may also be inappropriate to share, as in combination they can become identifying. For example, data collected from a
small group of participants, vulnerable populations, or private groups should not be shared if they involve indirect identifiers (such as sex, ethnicity, location,
etc.) that may risk the identification of study participants.
Additional guidance on preparing raw data for publication can be found in our Data Policy (https://journals.plos.org/plosone/s/data-availability#loc-humanresearch-participant-data-and-other-sensitive-data [journals.plos.org]) and in the following article: http://www.bmj.com/content/340/bmj.c181.long [bmj.com].
Please remove or anonymize all personal information (<up-id>), ensure that the data shared are in accordance with participant consent, and re-upload a fully
anonymized data set. Please note that spreadsheet columns with personal information must be removed and not hidden as all hidden columns will appear in the
published file.
Thank you for this information, but there appears to be a fundamental mis-understanding of the data in the S1-File. These are not related to any individual, rather they are computed data for 20503 proteins of the human proteome. They *do not* relate to person, patient, or private data. They *do* relate to proteins, each given by their UniProt identifier. I would be pleased to provide any further clarification that is required.

4. If the reviewer comments include a recommendation to cite specific previously published works, please review and evaluate these publications to determine
whether they are relevant and should be cited. There is no requirement to cite these works unless the editor has indicated otherwise.
[Note: HTML markup is below. Please do not edit.]
Reviewers' comments:
Reviewer's Responses to Questions
4
Comments to the Author
1. Is the manuscript technically sound, and do the data support the conclusions?
The manuscript must describe a technically sound piece of scientific research with data that supports the conclusions. Experiments must have been conducted
rigorously, with appropriate controls, replication, and sample sizes. The conclusions must be drawn appropriately based on the data presented.

Reviewer #1: Partly
Reviewer #2: Yes
2. Has the statistical analysis been performed appropriately and rigorously?
Reviewer #1: Yes
Reviewer #2: Yes
3. Have the authors made all data underlying the findings in their manuscript fully available?
The PLOS Data policy [track.editorialmanager.com] requires authors to make all data underlying the findings described in their manuscript fully available
without restriction, with rare exception (please refer to the Data Availability Statement in the manuscript PDF file). The data should be provided as part of the
manuscript or its supporting information, or deposited to a public repository. For example, in addition to summary statistics, the data points behind means,
medians and variance measures should be available. If there are restrictions on publicly sharing data—e.g. participant privacy or use of data from a third
party—those must be specified.
Reviewer #1: Yes
Reviewer #2: Yes
5
4. Is the manuscript presented in an intelligible fashion and written in standard English?
PLOS ONE does not copyedit accepted manuscripts, so the language in submitted articles must be clear, correct, and unambiguous. Any typographical or
grammatical errors should be corrected at revision, so please note any specific errors here.
Reviewer #1: Yes
Reviewer #2: Yes
5. Review Comments to the Author
Please use the space provided to explain your answers to the questions above. You may also include additional comments for the author, including concerns
about dual publication, research ethics, or publication ethics. (Please upload your review as an attachment if it exceeds 20,000 characters)

**Reviewer #1:** The manuscript entitled “Computational investigation suggests that the cell adhesion sub-proteome is enriched for sites of pH-dependence and
charge burial, whilst some key intracellular pathways may be shielded from pH fluctuations.” Research work done by Shalaw et al., utilizes computational
tools to investigate the pH predictions of computational models which detecting pH dependence and physiologically aspects of proteins in relevant human.
Exploring the characteristics of buried ionisable groups, with pK, pH charge networks might have high value predicted pH-dependence and/or charge
networking of proteins, are interesting and more valuable.
--The approach taken was fascinating and excellent. In order to define the subset of enzymes and transporters (ET) and non-enzymes and transporters (NET)
that contain the BCOI feature, which indicates a predicted pH-dependent function, the authors have effectively used the computational methods SASA (sacalc),
beta-carotene oxygenase 1 (BCO1), and pKa (pkcalc, PROPKA3).
-However, because AlphaFold2 protein models naturally alternate between different conformations based on their oligomeric state, AlphaFold may need to be
run with different settings or multiple predictions. The authors also run AlphaFold2 protomer models, which exhibit poor performance and some limitations.

We thank Reviewer for the kind observations about our work (a “fascinating and excellent” approach). We agree that using AlphaFold protomer models implies restrictions about the scope of the study. We have now expanded upon our comments on this, at the end of section 3.4, bringing in references to state of the art protein-protein interaction predictions, and why these do indeed represent the next challenging step for a proteome-wide analysis of pH-dependence. Predictions of interactions give models for interfaces, at different confidence levels, with the numbers of interfaces varying significantly as the confidence level changes. We show, in outline, that sequence conservation can be used to put likely bounds on the numbers of amino acids involved in functional pH-dependence, and aid future studies that go beyond the AlphaFold protomer analysis.

-The methods were explained in a vague manner, and the corresponding results were discussed in less interactive way.
However, the paper has some merits. Beyond the potentiality of the manuscript, I have a few concerns that the authors may address.

Major:

1.Can you confirm that the CamSol approach, which determines a protein's solubility based on the physicochemical characteristics of its amino acid sequences,
may be more accurate and dependable for proteins that are inherently disordered?

Thank you for the suggestion, we have added a reference to the use of PROPKA within the CamSol scheme for protein solubility prediction, in a pH-dependent version (near the end of the Introduction).

2. Why authors selected Semaphorins specifically not mentioned, Desmosomes calcium binding sites in
cadherin domains visualization missing, which are the molecular focus of BCOI prediction.
With regard to semaphorins, we realise that this requires clarification, and have now specified the Gene Ontology categories that directly reference semaphorins in the text at the start of section 3.6.3, and to which GO classification (component, process, function) and figure they refer.

With regard to desmosomes, we look at 8 different systems in separate sub-sections of section 3.6, 4 we describe in greater detail, including figures, and 4 are briefer and without figures. We have limited information for desmosomes, which is the shortest section, we have not added a figure, but have expanded a sentence at the end of 3.6.5 to previous work in which we have considered under what circumstances pH can couple to calcium binding.

3. figure legend appeared in middle if the manuscript is unusual PDB statures acronyms missing.
For the figure legends in the middle of manuscript, our understanding is that this conforms to PLoS One submission policy for review, and that otherwise would not conform.

In respect of PDB identifiers in Figures, Figure 9 includes these (in the Figure panels). For Figures 10 and 11, we had specified the PDB code (or AlphaFold model) in the Figure Legends. This has now been supplemented with PDB codes (colour-coded accordingly) also in the Figure panels, as requested.

Minor:

1. Figure legends labels need to be addressed, detailed explanation needed.

The Reviewer suggests several specific Figure updates, and we believe these have been addressed, in regard to their points that follow.

2. Can you highlight the amino acid residues on semaphorins charged networks 3-D structure figure models (Figure 9).
3. Same with the other models like Figure 9B plexins charge networks highlight the residual interactions maybe more reliable to readers.

These (Figure 9A and 9B) are composite diagrams of amino acids predicted to be in charge networks, averaging 10 residues for each semaphorin domain included (in either semaphorins or plexins). Inclusion of these would overwhelm the main point of these Figure panels, to show the locations of residues within the semaphorin domains. The Reviewer makes a good point though about revealing the identity of the residues. Our S1 File supplementary data contains all predicted BCOI for all human proteins, and therefore provides this information. A note to this effect has been added in section 3.6.3. We learn from the PLoS One comments earlier in this communication that our S1 File did not make it through to the original review process since it was falsely recognised as potentially containing patient data. It is purely about proteins and charges. Having clarified this, the S1 File should now be available, with the detail of amino acid predictions for each protein.

4. 8bf4 mouse plexin B1 with VHH14 and VHH15 in figure files can apply different colors for the (VHH14 and VHH15) will be better understanding.

Figure 9 uses the term “from” within the Figure to denote that only the plexin component is displayed from a complex. We should have stated this explicitly in the Figure legend, and have now done so. Thus the nanobodies VHH14 and VHH15 are not included in the Figures, since the intention is to highlight properties of the plexin.

Consider the more relevant studies in PMID: 35247105; PMID: 35301897 are more value added to this manuscript.

Both of these reference suggestions are now included in a new sentence at the end of section 3.6.2, that emphasises the importance of GPCRs in disease and response to injury (i.e. in addition to normal physiological function).

**Reviewer #2:** This manuscript presents a comprehensive and ambitious computational study that leverages AlphaFold2 models and pKa prediction methods to
analyze pH-dependence and charge burial across the human proteome. The core finding is novel, significant, and will be of broad interest to the fields of
computational biology, biophysics, and cell signaling. The methodology is generally sound, the scale of the analysis is impressive, and the study provides a
valuable resource for the community. However, there are several aspects require clarification and further analysis to fully support the claims and ensure the
robustness of the conclusions.

1.The title of this manuscript is quite long and syntactically complex, an accurate, clear and concise title is more recommended.
We have followed this suggestion and reduced the title to:

Computation suggests that the cell adhesion sub-proteome is enriched for sites of pH-dependence and charge burial.

This concentrates on the key message.

2. The restriction to AlphaFold2 protomer models is a significant limitation, and is vividly illustrated by the failure to predict key pH-sensing residues located
at oligomeric interfaces (e.g., in ASIC1/2, TRPV1). While the benchmark performance is good, the discussion should more forcefully address the potential for
systematic false negatives in certain protein classes (e.g., ion channels, transcription factors) due to this limitation.

A substantial discussion has been added at the end of the benchmarking section (3.4), stating that false negatives for predicting charges of interest are likely to be enriched for proteins that form interfaces (including transcription factors, channels and transporters). We also add a small analysis using AlphaMissense values to address questions of whether the predicted BCOI set makes sense, and also how large the number of groups excluded (based on the protomer analysis) might be. For the benchmark data in section 3.4, AlphaMissense scores largely support the BCOI predictions (scores are added to Table 2), and suggest an approximate threshold for extending beyond the benchmark set once interface structures are considered. A short discussion of interface modelling is also included, to indicate the direction that subsequent work will proceed. Interface discovery and modelling itself is incremental, fractionally through the human proteome. With a substantial set of charges of interest already predicted from protomer analysis, it is argued that using these in GO estimation of classes enriched for pH-dependence is reasonable.

3.Definition and Justification of BCOI Filters: The study employs ten different filters to define a "Buried Charge of Interest" (BCOI). While this multi-faceted
approach is a strength, the rationale for choosing the specific thresholds (e.g., SASA ≤ 15 Å², ΔpKa ≥ 2, network size ≥3) needs to be more explicitly justified.
A supplementary table summarizing the performance (sensitivity, specificity) of each filter against the benchmark set of 23 proteins would greatly help the
reader understand the relative strengths and weaknesses of each method.

These data have been compiled in a new S1 Table, which is introduced at the end of Section 3.2. Included are the filters depicted in Fig 1, so that the reader can see how the chosen 10 filters compare in terms of performance with respect to various subsets. This includes the benchmark set of 23 (pH-dependent) proteins, but does not include a sensitivity/specificity analysis for them since they constitute positive and unlabelled data. The ET *versus* NET distinction is reasonably well established from annotation, and that is used to generate sensitivity, specificity and balanced accuracy for the filters.

4.The GO analysis is a highlight of the paper. However, the biological interpretation of the charge networks in certain enriched categories (e.g., semaphoring domains, integrator complex) is currently speculative. The authors appropriately suggest roles in conformational buffering or mechanosensation beyond pHsensing. This discussion should be tightened to more clearly distinguish between data-driven observation (enrichment of BCOI) and hypothesis-driven speculation (potential functional role).

We accept the Reviewer’s comments, and recognise the step to speculation when discussing potential functional implications. Text at the end of section 3.6.3, near the end of section 3.6.8, and at the end of the Conclusion section, has been updated to emphasise that discussion of potential links to mechanotransduction and osmo-sensing are hypothesis only.

5. GO analysis revealed that BCOI is enriched at the cell periphery (particularly in cell adhesion molecules, ion channels, GPCRs), while it is depleted in
categories such as ribosomal and nuclear structures. These findings suggest that certain key intracellular processes may possess a "buffering" capacity against
pH fluctuations. Whether these enrichment/depletion patterns are related to subcellular pH environments or functional demands?

This is an interesting point. We have added some further discussion at the end of section 3.6. Our preference of subcellular pH *versus* functional demands is the latter since in some cases it is specific GO categories that are particularly enriched or depleted. The mitochondrion is a clear exception (entire organelle GO category strongly returned as depleted across filters), but here we speculate that it could be related to maintenance of the mitochondrial pH gradient.

6.In Figure 7 (GO Enrichment/Depletion)，the labels are illegible. A higher-resolution version of Figure 7 with clearly readable text is recommended. The
supporting figures (S1, S2) should also be briefly described in the main text to ensure readers do not overlook them.

We had spent some time during original submission trying to generate a Fig 7 that would maintain legibility during the PLoS One pdf conversion. We thought that we had just got it over the (legibility) line, but recognise the issue mentioned by the Reviewer. The Figure passes the PLoS online checker, and the downloadable version is high resolution and easily legible. It is just the PLoS One pdf conversion that seems to have an issue, whilst other pdf converters do not. We will try again, apologies for inconvenience caused.

With regard to S1 and S2 Figures, we now discuss these a little more at their Introduction in section 3.6.

7.Table 2 is very helpful. Consider adding a column for the SASA value of the reported pH-sensing residue in the protomer model to help readers contextualize
the prediction failures.

An SASA column has been added to Table 2, as suggested, and accompanying text is added towards the end of section 3.4.

8.Please expand slightly on what is meant by "functional resilience" in this context.

We have expanded, so that the sentence (in the Conclusions section) now reads:

“Alternatively, perhaps depletion of BCOI indicates that certain processes have evolved reduced sensitivity to fluctuations in pH, so that cellular structure and function associated with those processes is not substantially changed during pH fluctuations.”

6. PLOS authors have the option to publish the peer review history of their article (what does this mean? [track.editorialmanager.com]). If published, this will
include your full peer review and any attached files.
If you choose “no”, your identity will remain anonymous but your review may still be made public.
Do you want your identity to be public for this peer review? For information about this choice, including consent withdrawal, please see our Privacy Policy
[track.editorialmanager.com].
Reviewer #1: No
Reviewer #2: No
[NOTE: If reviewer comments were submitted as an attachment file, they will be attached to this email and accessible via the submission site. Please log into
your account, locate the manuscript record, and check for the action link "View Attachments". If this link does not appear, there are no attachment files.]
While revising your submission, please upload your figure files to the Preflight Analysis and Conversion Engine (PACE) digital diagnostic
tool, https://pacev2.apexcovantage.com/ [track.editorialmanager.com]. PACE helps ensure that figures meet PLOS requirements. To use PACE, you must first
register as a user. Registration is free. Then, login and navigate to the UPLOAD tab, where you will find detailed instructions on how to use the tool. If you
encounter any issues or have any questions when using PACE, please email PLOS at figures@plos.org. Please note that Supporting Information files do not
need this step.
